# Evaluation of a Ti–Base Alloy as Steam Cracking Reactor Material

**DOI:** 10.3390/ma12162550

**Published:** 2019-08-10

**Authors:** Stamatis A. Sarris, Kim Verbeken, Marie-Françoise Reyniers, Kevin M. Van Geem

**Affiliations:** Department of Materials, Textiles and Chemical Engineering, University of Gent, Technologiepark 914, BE-9052 Zwijnaarde, Belgium

**Keywords:** cracking, coke, superalloy, carburization, oxidation

## Abstract

Low-coking reactor material technologies are key for improving the performance and sustainability of steam crackers. In an attempt to appraise the coking performance of an alternative Ti–base alloy during ethane steam cracking, an experimental study was performed in a jet stirred reactor under industrially relevant conditions using thermogravimetry (T_gasphase_ = 1173 K, P_tot_ = 0.1 MPa, X_C2H6_ = 70%, and dilution δ = 0.33 kg_H2O_/kg_HC_). Initially, a typical pretreatment used for Fe–Ni–Cr alloys was utilized and compared with a pretreatment at increased temperature, aiming at better surface oxidation and thus suppressing coke formation. The results revealed a decrease in coking rates upon high temperature pretreatment of the Ti–base alloy, however, its coking performance was significantly worse compared to the typically used Fe–Ni–Cr alloys, and carbon oxides formation increased by a factor of 30 or more. Moreover, the analyzed coupons showed crack propagation after coking/decoking and cooling down to ambient temperature. Scanning electron microscopy combined with energy-dispersive X-ray spectroscopy indicated that the prompt and unsystematic oxidation of the surface and bulk caused observable crack initiation and propagation due to alloy brittleness. Hence, the tested Ti–base alloy cannot be considered an industrially noteworthy steam cracking reactor alloy.

## 1. Introduction

The predominant process for manufacturing light olefins is steam cracking of hydrocarbons [1]. Coking of the used materials in different parts of the cracker is one of the key problems of this process. The generated coke is formed on the inner surface and reduces the available cross-sectional area causing an elevated pressure drop along the reactor, convection section, and Transfer Line Exchanger (TLE). Higher average pressures decrease the light olefin’s selectivity because they promote bimolecular over monomolecular reactions. As a result, more energy and feed is required for maintaining the crackers’ production capacity, resulting in substantial financial losses [2,3]. When the metallurgic limits are reached or the pressure drop over the reactor is excessive, the operation is stopped and decoking of the reactor is necessary, typically lasting 48 h. The latter further affects the profit margin [4]. In an effort to mitigate coke formation, three coke reducing technologies are currently used, namely three dimensional (3D) reactor technologies [5,6,7,8], application of additives [9,10,11,12,13,14,15,16,17,18,19,20,21], and use of advanced material technologies [22,23,24,25,26,27,28]. The first technology consists of, among others, the use of finned [5,8], ribbed [6], or partially ribbed tubes and swirl flow [7] tubes. All these technologies lead to an increased pressure drop compared to conventional bare tubes, and hence affect the ethene selectivity. The second category consists primarily of the application of sulfur-containing compounds [10,11,12,13,14,15,16,17,18,21]. The role of sulfur additives on diminishing carbon monoxide formation is well established, but their effect on coke formation is debated [9].

In this work, the focus is on novel materials for the reactor, TLE, and convection section. Minor and major changes in bulk alloy and/or surface composition can substantially alter the coking tendency. Steam cracking reactors are typically made out of heat-resistant Fe–Ni–Cr alloys (25/35 Cr/Ni) [22,23]. Aluminum and manganese are often added to enhance the coking resistance of the alloys by forming a protective alumina or a manganese chromite (MnCr_2_O_4_) spinel layer, respectively. The high cost of these materials is a strong driver for investigating cheaper alternatives such as titanium-base alloys. Titanium and titanium alloys are used [29] for aerospace applications because of their high strength to weight ratio and excellent corrosion resistance. Furthermore, titanium additions to Fe–Ni–Al–Cr ferritic alloys [30] is found to improve tensile strength, toughness, creep properties, and corrosion resistance [31]. Often, Ti is added in concentrations of 0–10 wt.% to a high temperature alloy [32], to increase rigidity. In addition, in catalytic steam reforming, the addition of Ti and V is known to suppress the generation of amorphous coke, promoting graphitic coke formation, while it can suppress catalytic steam reforming [33]. In the process studied, steam cracking of hydrocarbons—a noncatalytic process—coke forms on the pretreated reactor material, which is traditionally [34] oxidized to inhibit carbon formation. Therefore, no a priori connection can be made between the effects of adding an element in the reactor material and its coking tendency. The anticoking performance of titanium oxide and TiC coatings in hydrocarbon pyrolysis has been reported previously [35]. However, the use of a Ti–base alloy in stream cracking reactors has not been evaluated yet. Therefore, in this work the coking performance of a Ti–base alloy (Ti–6Al–4V), under ethane steam cracking conditions, is studied using a thermogravimetric setup. Furthermore, the pretreatment temperature of the material is studied because the latter can significantly alter its coking performance [36]. The coking results are compared to a classically used Fe–Ni–Cr for reference. Scanning electron microscope and energy-dispersive X-ray (SEM and EDX) results help with the interpretation of the coking and product distribution.

## 2. Experimental Section

### 2.1. ElectroBalance Unit

**Figure 1:**
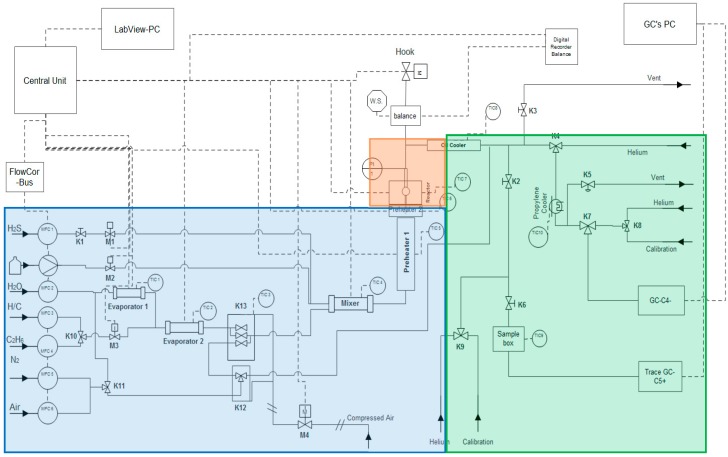
Simplified process flow diagram of the thermogravimetric setup for the study of coke deposition during steam cracking of ethane. (Feed section (blue), Reaction section (red), and Analysis section (green)).

The jet stirred reactor (JSR) setup has been developed and used extensively for many reactor material studies [22,23,36,37]. Thus, a concise explanation is provided and shown in Figure 1. The flow rates of gasses and water are controlled by thermal mass flow controllers. The water is vaporized in an evaporator and mixed with the heated hydrocarbon feedstock. They are then further heated before entering the reactor inlet at a temperature of 903 K. A quartz jet stirred reactor is used, with a rectangular coupon with dimensions of 10 mm × 8 mm × 1 mm. The coupon is suspended from the arm of an electrobalance in the center of the JSR (see Figure S1). In Figure S2, it can be seen that the mixing inside the reactor is quite ideal and therefore it is a suitable set-up for the study of coke formation on high temperature alloys. The electrobalance has the ability to measure mass changes over time with a frequency of 1 Hz. Subsequently, the reactor effluent is quenched to counteract further cracking. In order to analyze its composition, the reactor effluent is injected online in two gas chromatographs (GC) using nitrogen as internal standard, this acts as a refinery gas analyzer dedicated to the analysis of components with less than 5 carbon atoms and a TRACE^TM^ Ultra GC detecting hydrocarbons ranging from methane to naphthalene. For ethane cracking, no products heavier than naphthalene were identified in the effluent. In Figure S3 of the supporting information, a schematic representation of the use of the internal standard is provided. 

### 2.2. Experimental Procedures and Conditions

The coking performance of a new Ti–base alloy under ethane steam cracking conditions was evaluated in the JSR setup. In addition, the effect of the in situ pretreatment was also evaluated.

Two different pretreatments were used: a pretreatment that was previously optimized for Fe–Ni–Cr alloys, and one at a higher oxidation temperature. The experiments conducted in the JSR consist of in total 8 cracking cycles—3 cycles of 6 h coking followed by decoking, followed by four short cycles of 1 h cracking to cyclically age the coupon surface and decoking, and then a last cycle of 6 h—to mimic the effect of periodic cracking and decoking. This procedure is called cyclic aging. Table 1 and Table 2 summarize the experimental conditions and sequence of steps used in the two pretreatments. Cooling down and heating up is fixed to a rate of 300 °C per hour.

### 2.3. Surface Characterization

The SEM analyses of the coupons were performed using a JEOL analyzer, type JSM-7600F (University of Gent, Zwijnaarde, Belgium), with a Schottky field emission gun as an electron source. SEM and EDX were used to obtain information regarding the surface morphology and elemental composition of the samples. Both top surface and cross-section analyses of the coked samples were performed in this study. The top surface analysis gives a qualitative idea of the coke growth together with elemental quantitative data of the surface, obtained at 10 kV and 20 kV. The cross-section mappings allow examination of the surface oxide layer generated due to exposure to an oxidative or carburizing environment. Furthermore, the microstructure of the cyclically aged alloy is evaluated. In the EDX analysis, the detected oxygen and carbon are omitted given their limited accuracy with the used EDX.

### 2.4. Coke Formation Mechanisms

The carbon deposition process during thermal cracking in the presence or absence of steam, is quite a complex phenomenon. It has been extensively described in the literature [37,38,39,40,41] that it mainly consists of three mechanisms: catalytic coke formation, coke growth from existing carbon layers onwards (pyrolytic), and gas phase coking, as illustrated in Figure S4.

Initially, carbon deposition takes place and creates a porous network of carbon filaments, catalyzed mainly by Ni and Fe present at the surface of the reactor walls. It is widely accepted that catalytic carbon formation [42,43,44,45,46] on metallic surfaces involves surface reactions, diffusion, and precipitation of carbon. First, a hydrocarbon molecule is chemisorbed onto a metal crystallite of the surface. Dehydrogenation of R–CH groups takes place with the hydrogen atoms recombining and desorbing into the gas phase. As a result, carbon atoms are deposited on the surface, dissolve in, and diffuse through the metal particle. Carbon accumulation in the particle causes pressure build up at the dislocations and the grain boundaries, which may exceed the tensile strength of the metal.

Potentially, the metal particle is then lifted from the surface and carbon crystallizes at the rear end of the particle. Growing stems that carry crystallites on their top are developed. The precipitation of carbon can give rise to structural deficiencies in carbon lattice, thereby creating reactive carbon sites along this layer. Furthermore, hydrocarbon molecules from the gas phase are incorporated at these sites causing further coke growth. In that way, a porous layer of interwoven filaments is developed. The metal particles at the top of the whiskers become more accessible from the gas phase. Therefore, the carbon deposition rate increases, while the diffusion rate through the metal remains stable. Carbon migration over the metal surface occurs, surrounding the carbon stems. At this point, surface carbon can occur, encapsulating the metal thereby hampering further catalytic coke growth.

Dissociative chemisorption of water molecules on metal particles produces highly reactive oxygen atoms that react with surface carbon to from carbon monoxide, which desorbs to the gas phase. This reaction prevents the fast encapsulation of the filament’s metal tip, and therefore the termination of this reaction, in other words the “deactivation of the metallic active site”. How quickly these phenomena occur is a matter of relative kinetics of carbon growth, gasification, dissolution, and diffusion. The properties of the alloy play a role in these catalytic mechanisms, similar to the alloy evaluation presented in this work.

The heterogeneous noncatalytic mechanism is the major source of coke in an industrial cracker, since it takes place over the complete run length. The coke in contact with the gas phase looks like a succession of several discrete layers, deriving from the coke–gas interface [47,48]. Coke formation is described by the reaction of gas phase precursors with active centers of the surface. Kopinke et al. [38] provide a general scale for the coking tendency of several precursors. For example, acetylene, anthracenes, cyclic naphthenes, and aromatics possess a high tendency for coke formation. Ethylene is not the most reactive one, however, is very important due to its high concentration. No significant differences of the relative constants of a particular hydrocarbon were found for different materials [38]. These results support the fact that the active centers are radical in nature and positioned in the coke matrix. The radicals can be generated by hydrogen abstraction from the partially dehydrogenated carbon layer being determined by the gas phase composition and the available coke surface [49]. The coke radicals react with unsaturated molecules and radicals from the gas phase. After dehydrogenation, graphitic layers are formed [50].

The last mechanism, also known as homogeneous noncatalytic coking, results from a sequence of molecular and/or radical reactions in the gas phase. These lead to high molecular weight polynuclear aromatic compounds, which remain solid even at high temperatures. As a result, they collide at the wall and integrate in the coke layer [51]. In this work, the latter mechanism contributes very little, due to the small amounts of heavy compounds generated in the gas phase during cracking of the light feedstock (ethane).

## 3. Experimental Results

### 3.1. Product Yields and Coking Tendency of Ti–Base Alloys

#### 3.1.1. Fe–Ni–Cr Alloy versus Ti–Base Alloy

Table 3 illustrates the most important product yields during ethane cracking. For a better comparison, the product yields of a traditionally used Fe–Ni–Cr reactor material are also included. In previous work, pretreatment for this Fe–Ni–Cr material was studied and pretreatment A (see Table 2) was found to provide a surface oxide layer that exhibited a very good protection against coke formation. For the same pretreatment conditions, there are only some minor differences in product yield distribution between the reference Fe–Ni–Cr alloy and the Ti–base one. The only pronounced influence of the material was observed in the values of CO, where increase by a factor of 30 was noted. For CO_2_, the impact is less pronounced. As expected the increase in carbon oxides was accompanied by an increase in H_2_. It is important to note that at the same conversion, the yield of ethylene was also slightly lower for the Ti alloy. Although the difference seems minor, for a world scale unit this difference will have a substantial effect on profit margins.

Coking rates are summarized in Table 4. Based on the results, the Ti–base alloy generates at least three times more coke than the reference material, using pretreatment A.

#### 3.1.2. Pretreatment Effect

According to the literature [52,53,54,55], eight times increase in the absolute weight of the formed surface oxides on titanium alloys is expected by increasing the temperature from 1173 K to 1273 K. By increasing the oxidation temperature from 1173 K to 1273 K in pretreatment B (see Table 2) for the Ti–base alloy, there is an increase by a factor of 30 in the yield of CO compared to the reference material, and by a factor of 3 compared to the Ti–base alloy after application of pretreatment A. For the CO_2_ yield, an increase by a factor of 3 was observed. Similarly, the increase of carbon oxides is accompanied by an increase in H_2_.

**Figure 2:**
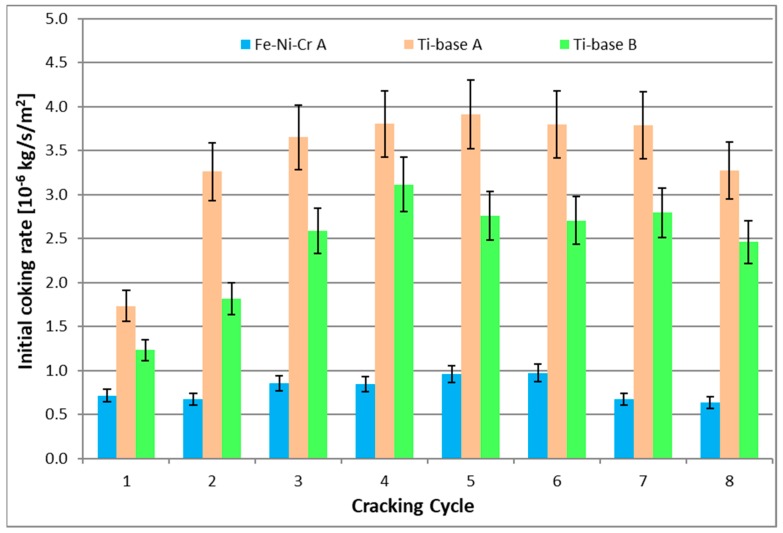
Calculated initial coking rates for the reference 25/35 Cr/Ni and Ti–base alloy. Ethane steam cracking: F_HC_ = 29.18 × 10^−6^ kg s^−1^, δ = 0.33 kg_H2O_ kg^−1^ HC, T_reactor_ = 1173.15 K, P = 101.35 kPa, F_H2O_ = 9.72 × 10^−6^ kg·s^−1^.

**Figure 3:**
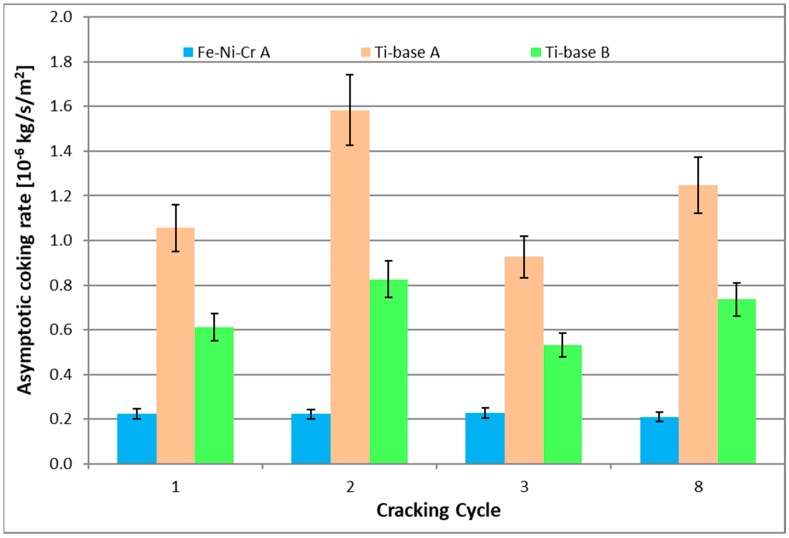
Calculated asymptotic coking rates for the reference 25/35 Cr/Ni and Ti–base alloy. Ethane steam cracking: F_HC_ = 29.18 × 10^−6^ kg s^−1^, δ = 0.33 kg_H2O_ kg^−1^ HC, T_reactor_ = 1173.15 K, P = 101.35 kPa, F_H2O_ = 9.72 × 10^−6^ kg·s^−1^.

As it is observed in Figure 2 and Figure 3, independently on the cracking cycle, the Ti–base alloy performs significantly worse than the reference material. The increased temperature pretreatment (B) improves the coking rates of the Ti–base alloy. However, this improvement in coking behavior is still significantly worse compared to the coking behavior on the reference Fe–Ni–Cr alloy.

Carbon oxides are produced by gasification of coke with steam and/or by steam reforming. The observed increase in carbon oxides suggests that Ti and/or Ti oxides present on the surface have a catalytic effect on coke gasification in view of the fact that Ti has been reported to suppress steam reforming [33]. Since large amounts of CO in the effluent can cause problems in the downstream separation section of an industrial steam cracker, these data indicate that the Ti–base alloy performs significantly worse than the reference material.

In addition, the coking behavior remains rather stable during cyclic aging for the reference material, while the Ti–base alloy shows increased coking after several cycles. Independent of the pretreatment applied, coke deposition on the Ti–base alloy shows ~40% increase with aging. Since industrial crackers typically require decoking after 2–3 months, the observed increase in coking after cyclic aging of the Ti–base alloy strongly limits its potential as a construction material for industrial steam crackers [30].

### 3.2. Evaluation of Ti–Base Alloy Using SEM and EDX Analysis

The most problematic result obtained with the Ti–base alloy coupons was their behavior after cooling down to ambient conditions. More specifically, the coupons broke either into slices in the case of pretreatment A or into smaller pieces in the case of the coupons oxidized with pretreatment B. In Supporting Information Figure S5, an indication regarding the coupon thickness decrease after cracking and the thickness of the slices is given. The total coupon thickness before cracking and before breakage is around 1 mm, while by adding the slice thickness and coupon thickness after cracking, it exceeds 1.1 mm. Clearly, thick oxides are formed during oxidation of Ti—almost 10% of the total coupon volume—making the coupon more brittle and more sensitive to mechanical stresses. The latter leads to crack formation especially during cooling down to temperatures below 1023 K. Crack formation allows access of reactant gases to the untreated bulk material areas, exposing the catalytic surface that is not passivated by the pretreatment.

An example of a cracked coupon, that underwent pretreatment B, is shown in the supporting information in Figure S6, where the breakage occurred diagonally, leading to smaller coupon pieces. To illustrate crack formation on the coupons, and for a better understanding of the phenomenon, SEM and EDX analyses were performed. 

#### 3.2.1. Decoked Samples

**Figure 4:**
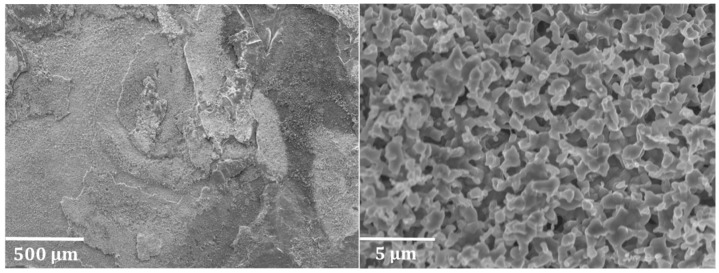
Decoked Ti–base alloy coupon after application of pretreatment A and eight coking/decoking cycles using pretreatment A. Accelerating voltage of 10 kV.

Figure 4 shows a decoked Ti–base alloy sample that underwent pretreatment A. The sample was exposed to eight cracking cycles and was decoked in the last cycle. It is clear that the oxides have spalled off during the cooling down and removal of the coupon from the reactor. Fracture areas can be observed, and the surface displays an irregular depth profile. The surface shows signs that the oxide layer has spalled off from the metal’s surface.

**Figure 5:**
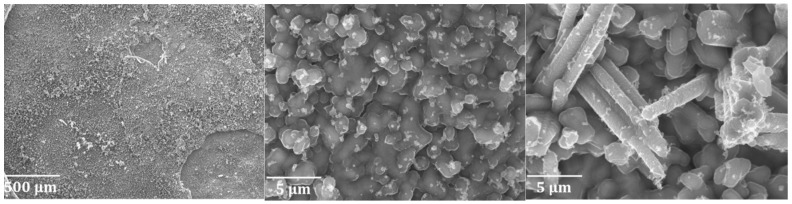
Surface of a decoked Ti–base alloy after pretreatment B and two coking/decoking cycles at an accelerating voltage of 20 kV.

Figure 5 shows the surface of a decoked Ti–base alloy coupon that underwent pretreatment B and two cracking cycles and decoking. The left image in Figure 5 shows a surface on which spalling of the oxide layer has occurred in some areas. The picture in the middle of Figure 5 shows an area where spalling did not take place, while the picture on the right of Figure 5 shows the area where spallation has occurred. Clearly, spalling off from the oxide layer has an effect on the formed surface structure and is possibly responsible for the pronounced coking of the Ti–base alloy upon cyclic aging. 

#### 3.2.2. Coked Samples

**Figure 6:**
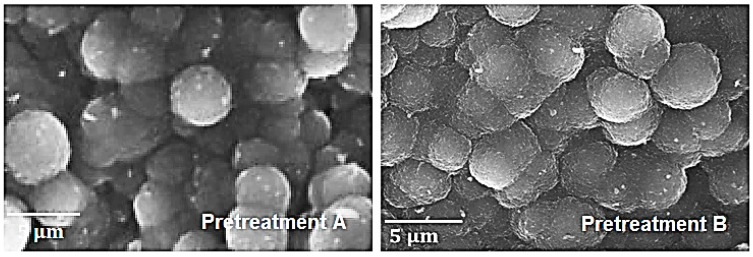
Top surface of the coked Ti–base alloy after application of pretreatment A and pretreatment B, and seven coking/decoking cycles followed by a 6 h coking at an accelerating voltage of 20 kV.

Figure 6 illustrates the top surface of the coked Ti–base alloy coupons after seven coking/decoking cycles followed by a cracking cycle lasting 6 hours in the case of pretreatment A and B. In both pictures, the observed structures appear similar, implying that the coking mechanism was not different after applying two different pretreatments. 

**Figure 7:**
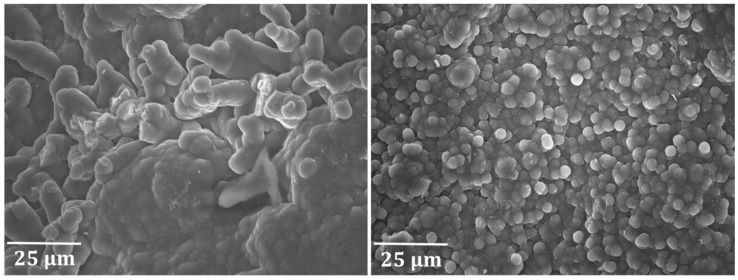
Coke structures recorded at an accelerating voltage of 20 kV on the top surface of the Ti–base alloy after applying pretreatment A and seven coking/decoking cycles followed by a 6 h coking.

Figure 7 shows the coke structures formed on the Ti–base alloy after applying pretreatment A and seven coking/decoking cycles followed by a 6 h coking. On the left, the formation of filamentous coke is noted, while on the right areas exist with a pronounced presence of nodular coke. Coke coverage of the Ti–base alloy is not homogeneous. It can be remarked that the typical nodular coke structures identified on Fe–Ni–Cr alloy samples are similar to those found on titanium alloys. However, in some areas filamentous coke is also noted, indicating the catalytic behavior of the Ti–base alloy.

**Figure 8:**
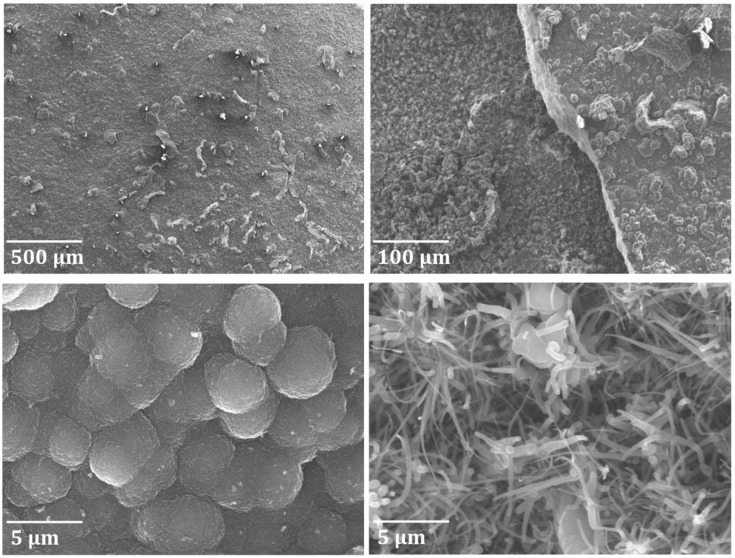
Surface of a coked Ti–base alloy coupon after pretreatment B and seven coking/decoking cycles followed by a 6 h coking. The figure on the top left shows the surface at a magnification of 50×, while the figure on the top right shows the area of breakage at a magnification of 100×. Both images at the top are recorded at an accelerating voltage of 10 kV. The image on the bottom left displays the regular surface area, while the image on the bottom right shows the area where breakage of the coupon occurred. Both images at the bottom are recorded at an accelerating voltage of 20 kV.

Lastly, a coked Ti–base alloy coupon after pretreatment B and seven coking/decoking cycles followed by a 6 h coking is represented in Figure 8. The picture on the top left of Figure 8 shows the irregular surface structure with titanium fragments, which have a lighter color. This coupon was broken into pieces upon extraction from the JSR reactor. The high titanium content of the alloy gives strength to the material, but also makes it more prone to fractures. High strength is inevitably associated with a certain brittleness. The image on the top right of Figure 8 displays an area where fracturing occurred and the coupon broke into two parts. A substantial height difference between the two areas of fracture can be observed. The image on the bottom left of Figure 8 shows the regular surface area with nodular coke formation. The formation of this type of coke is in accordance with the observations regarding the two other coupons. However, on the bottom right of Figure 8, an image is shown where breakage of the coupon took place. An entirely different structure can be observed in this image (Figure 8, bottom right) showing that at the breakage point more whiskers and filaments are noticed, illustrating the exposure of fresh catalytic sites due to crack formation.

#### 3.2.3. Cross-Sectional Analyses

**Figure 9:**
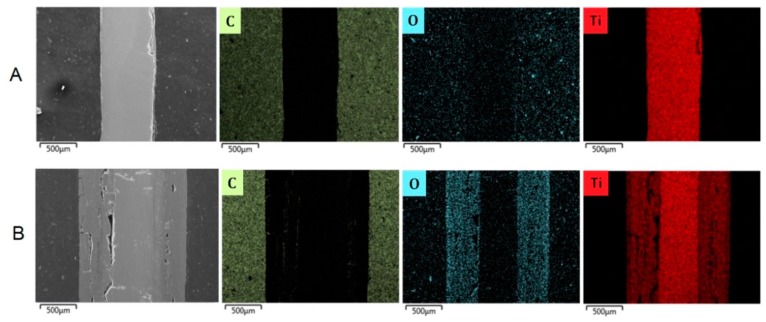
SEM and EDX cross-sectional analyses of coked Ti–base alloy samples after pretreatment and seven coking/decoking cycles followed by a 6 h coking. Accelerating Voltage: 15 kV. A refers to pretreatment A and B to pretreatment B.

**Figure 10:**
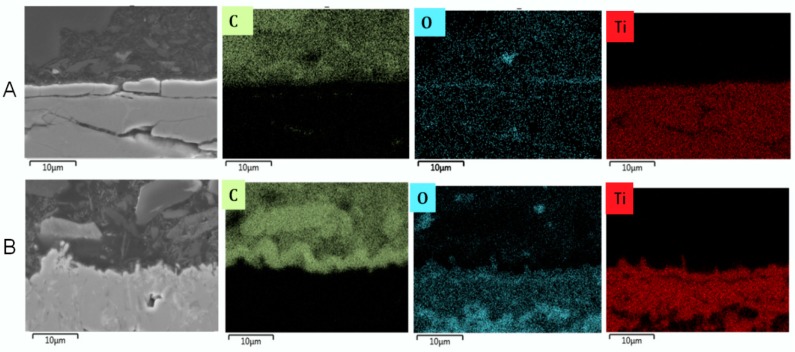
SEM and EDX cross-sectional analysis of coked Ti–base alloy samples after pretreatment and seven coking/decoking cycles followed by a 6 h coking. Accelerating Voltage: 15 kV. A refers to pretreatment A and B to pretreatment B.

Cross-sectional analyses of the two coked samples pretreated at two different temperatures revealed part of the phenomenon occurring in this study (Figure 9 and Figure 10). At a pretreatment temperature of 1173 K (pretreatment A), 0.1–0.2 μm thick oxides are formed that have spalled off the coupon leaving no oxide covering the surface after cooling down to ambient temperature. As a result, the coupon is significantly thinner than the fresh one. At a pretreatment temperature of 1273 K (pretreatment B), thick oxides covering 2/3 of the coupon body are formed leading to oxidation of almost the whole coupon volume. Clearly the oxides that are formed are quite brittle, and are separating from the core of the coupon. In the case of pretreatment B, the remaining core is rather thin and crack propagating can cause breakage of the coupon into smaller pieces or slices. Figure 10, a zoom in of Figure 9, clearly illustrates that oxidation at 1173 K (pretreatment A) results in a very weakly adhering oxide layer that can easily spall off from the coupon, while at higher temperatures (pretreatment B), extensive surface and bulk oxidation occurs.

**Figure 11:**
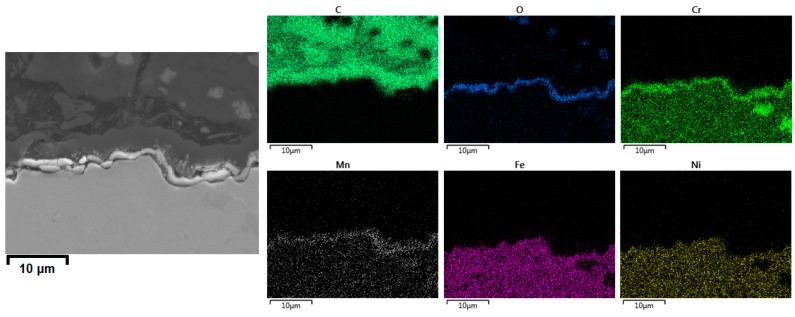
SEM and EDX cross-sectional elemental mappings of the coked Cr–Ni–Fe reference alloy after pretreatment A and seven coking/decoking cycles followed by a 6 h coking. Accelerating Voltage: 15 kV.

Based on Figure 10 and Figure 11, we can compare the Ti–base alloy with the reference Fe–Ni–Cr alloy. It is evident that on the reference material, a thin, uniform, and well-adhering manganese chromite (MnCr_2_O_4_) spinel layer is formed, providing an efficient suppression of coke formation [36]. In contrast, disorderly oxidation of the surface and bulk made the Ti–base alloy brittle, leading to observable crack initiation and propagation. Furthermore, the increased coking upon cyclic aging and the increased values of carbon oxides and H_2_ formation observed for the Ti–base alloy suggest that oxide spalling and/or crack formation result in exposure of the fresh catalytic sites. As a result, both coke formation and coke gasification were catalyzed using the Ti–base alloy.

## 4. Conclusions

A new Ti–6Al–4V alloy has been evaluated as a potential material for steam cracker reactors. The coke formed on the alloy under industrially relevant ethane steam cracking conditions was measured in a jet stirred reactor equipped with an electrobalance. Compared to the commonly used Cr–Ni–Fe alloy, CO formation increased by a factor of 30 yield, while no significant changes in other product yields were measured over eight cracking cycles. Upon increasing the preoxidation temperature from 1173 K to 1273 K, the CO_2_ yield increased by a factor of 3 compared to the Fe–Ni–Cr alloys preoxidized at 1173 K. The increased formation of carbon oxides is accompanied by an increase of the H_2_ yield, suggesting that the oxidized Ti–base alloy is more active towards catalytic coke gasification. Furthermore, coking rates on the Ti–base alloy are significantly higher and significantly increase upon cyclic aging.

Material failure of the Ti–base alloy coupons upon cooling down and removal from the reactor was observed. SEM and EDX revealed that upon preoxidation of the Ti–base alloy at 1173 K, a weakly adhering oxide layer of 0.1–0.2 μm is formed while at 1273 K significant bulk oxide formation leads to increased embrittlement of the material. Increased coke formation upon cyclic aging, and the increased formation of carbon oxides can be due to spalling off from the oxide layer and/or crack initiation and propagation resulting in exposure of fresh catalytic sites.

Hence, it can be concluded that use of the Ti–base alloy in industrial crackers poses high risks while offering low gain, and is definitely not beneficial in terms of coking behavior and the formation of carbon oxides.

## Figures and Tables

**Table 1:** Conditions of the two different applied pretreatments.

Pretreatment Id	Steps		
	1	2	3
A	6.7 × 10^−3^ Nl s^−1^ of Air; 14 h; T = 1023 K	1:1 8.15 × 10^−3^ Nl s^−1^ of Air:N_2_; 30 min; T = 1023–1173 K	8.15 × 10^−3^ Nl s^−1^ of Air and 6.67 × 10^−6^ kg s^−1^ Steam; 15 min; T = 1173 K
B	6.7 × 10^−3^ Nl s^−1^ of Air; 14 h; T = 1023 K	1:1 8.15 × 10^−3^ Nl s^−1^ of Air:N_2_; 30 min; T = 1023–1273 K	8.15 × 10^−3^ Nl s^−1^ of Air and 6.67 × 10^−6^ kg s^−1^ Steam; 15 min; T = 1273 K

**Table 2:** Experimental sequence during the two performed pretreatments.

**Pretreatment A**											
**Temperature**	**1023 K**	**1023–1173 K**	**1173 K**	**1173 K**	**1023–1173 K**	**1173 K**	**1173 K**	**1023–1173 K**	**1173 K**	**1173–273 K**	
Feed	Air	N_2_ + Air	Air + H_2_O	C_2_H_6_ + H_2_O	N_2_ + Air	Air + H_2_O	C_2_H_6_ + H_2_O	N_2_ + Air	Air + H_2_O	He	
Step	In situ Preoxidation 14 h	mild Preoxidation 30 min	Steam Treatment 15 min	1st coking cycle of 6 h	Decoking 30 min	Steam Treatment 15 min	2nd–8th coking cycle of [2nd, 3rd, and 8th of 6 h, 4–7th of 1 h]	Decoking 30 min	Steam Treatment 15 min	Cooling down	time
**Pretreatment B**											
**Temperature**	**1023 K**	**1023–1273 K**	**1273 K**	**1173 °C**	**1023–1273 K**	**1273 K**	**1173 K**	**1023–1273 K**	**1273 K**	**1273–273 K**	
Feed	Air	N_2_ + Air	Air + H_2_O	C_2_H_6_ + H_2_O	N_2_ + Air	Air + H_2_O	C_2_H_6_ + H_2_O	N_2_ + Air	Air + H_2_O	He	
Step	In situ Preoxidation 14 h	High T Preoxidation 50 min	Steam Treatment 15 min	1st coking cycle of 6 h	Decoking at higher T 50 min	Steam Treatment 15 min	2nd–8th coking cycle of [2nd, 3rd, and 8th of 6 h, 4–7th of 1 h]	Decoking at higher T 50 min	Steam Treatment 15 min	Cooling down	time

**Table 3:** Averaged (over the eight cracking cycles) mass product yields.

Components [wt.%]	Fe–Ni–Cr Alloy	Ti–Base Alloy	Ti–Base Alloy
Pretreatment	A	A	B
H_2_ [±0.07]	4.24	4.39	4.45
CO_2_ [±0.003]	0.006	0.005	0.019
CO	0.05 ^1^	0.56 ^2^	1.87 ^3^
CH_4_ [±0.21]	6.99	6.97	6.91
C_2_H_6_ [±0.52]	30.13	30.19	30.06
C_2_H_4_ [±0.25]	49.86	49.61	49.57
C_3_H_8_ [±0.03]	0.11	0.11	0.11
C_3_H_6_ [±0.02]	0.74	0.70	0.69
C_2_H_2_ [±0.05]	1.38	1.46	1.41
1,3-C_4_H_6_ [±0.07]	2.00	1.93	1.89
Benzene [±0.13]	2.46	2.53	2.53

Ethane steam cracking: F_HC_ = 29.18 × 10^−6^ kg s^−1^, δ = 0.33 kg_H2O_ kg^−1^ HC, T_reactor_ = 1173.15 K, P = 101.35 kPa, F_H2O_ = 9.72 × 10^−6^ kg·s^−1^; ^1^ [±0.008], ^2^ [±0.07], and ^3^ [±0.35].

**Table 4:** Calculated initial (average 15–60 min) and asymptotic (average 5–6 h) coking rates.

Alloy	Fe–Ni–Cr Alloy	Ti–Base Alloy	Ti–Base Alloy
Conditions	Blank	Blank	Blank
Pretreatment	A	A	B
Cracking Temperature (K)	1173	1173	1173
dilution	0.33	0.33	0.33
cc	Coke Formed (mg)		
1	1.29	5.48	3.66
2	1.26	9.01	4.99
3	1.41	7.43	4.82
4	0.60	3.57	2.93
5	0.67	3.03	2.17
6	0.69	2.97	2.13
7	0.47	2.95	2.32
8	1.19	7.69	5.35
cc	Initial Coking Rate [10^−6^ kg/s/m^2^]		
1	0.71	1.73	1.23
2	0.67	3.26	1.81
3	0.86	3.65	2.59
4	0.85	3.80	3.11
5	0.96	3.91	2.76
6	0.97	3.80	2.70
7	0.67	3.79	2.79
8	0.63	3.27	2.46
cc	Asymptotic Coking Rate [10^−6^ kg/s/m^2^]		
1	0.22	1.06	0.61
2	0.22	1.58	0.83
3	0.23	0.93	0.53
8	0.21	1.25	0.74

Ethane steam cracking: F_HC_ = 29.18 × 10^−6^ kg s^−1^, δ = 0.33 kg_H2O_ kg^−1^ HC, T_reactor_ = 1173.15 K, P = 101.35 kPa, F_H2O_ = 9.72 × 10^−6^ kg s^−1^.

## Supplementary Materials

The following information is available online at https://www.mdpi.com/1996-1944/12/16/2550/s1. Figure S1: Coupon position in the reactor; Figure S2: Cross-section fields of temperature (a) and ethane mass fraction (b) for ethane cracking experiments in the JSR; Figure S3: Schematic overview of the use of internal standards for quantitative composition analysis; Figure S4: Comparison of the relative importance of the three main coking mechanisms; Figure S5: Thickness values of the Ti–base alloy before cracking (left), after cracking (middle), and of a broken slice (right), pretreated at 1173 K; Figure S6: Broken Ti–base alloy coupon after cracking pretreated at 1273 K.

## Nomenclature

A	atomic mass of element [g·mol^−1^]
EDM	wire-cut electrical discharge machining
EDX	energy dispersive X-ray analysis
FID	flame ionization detector
F	flow rate [10^−6^ kg s^−1^]
H	penetration depth [µm]
cc	Coking/decoking cycle number i
JSR	jet-stirred reactor
L	nominal thickness [μm]
m_tj_	mass of coke at time j [kg]
M_c_	amount of coke deposited on the sample [g]
P_tot_	reactor pressure [MPa]
P/E	ratio of propylene [wt.%] to ethylene [wt.%]
r_c_	coking rate [kg·s^−1^·m^−2^]
r_c, initial_	initial catalytic coking rate [kg·s^−1^·m^−2^]
r_c, asymptotic_	asymptotic coking rate [kg·s^−1^·m^−2^]
r_x-y_	average coking rate in between the given time intervals [kg·s^−1^·m^−2^]
RGA	refinery gas analyzer
S	surface area of the coupon [m^2^]
SEM	scanning electron microscopy
TCD	thermal conductivity detector
T	temperature [K]
V	accelerating voltage [kV]
z	atomic number
δ	dilution [kg·kg^−1^]
ρ	density of the material [g·cm^-3^]
τ	mean residence time [s]
