# Supplementary material for: Evaluation of a Ti–Base Alloy as Steam Cracking Reactor Material
Source: Materials (Basel). 2019 Aug 10;12(16):2550. doi: 10.3390/ma12162550 (PMC6719091; doi:10.3390/ma12162550)
Supplement: Supplementary file 1 [file materials-12-02550-s001.pdf]

Supporting Information

Evaluation of a Ti–base Alloy as Steam Cracking Reactor Material

Stamatis A. Sarris, Kim Verbeken, Marie-Françoise Reyniers and Kevin M. Van Geem *

Department of Materials, Textiles and Chemical Engineering, University of Gent, Technologiepark 914, BE-9052 Zwijnaarde, Belgium

* Correspondence: Kevin.VanGeem@ugent.be

Jet Stirred Reactor

A small coupon of dimensions 10 mm × 8 mm × 1 mm is hanged by the arm of the electro balance in the center of a spherical jet stirred reactor, right above the jets, as illustrated in Figure S1. As recently published by our research group and is represented in Figure S2 [1], the mixing is quite ideal, with negligible changes of the gas phase concentrations and temperature around the coupon. Therefore, the set-up is suitable for the study of coke formation on high temperature alloys.

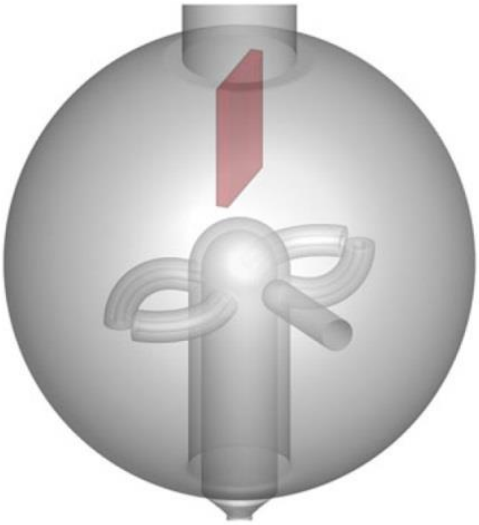

Figure S1. Coupon position in the reactor.

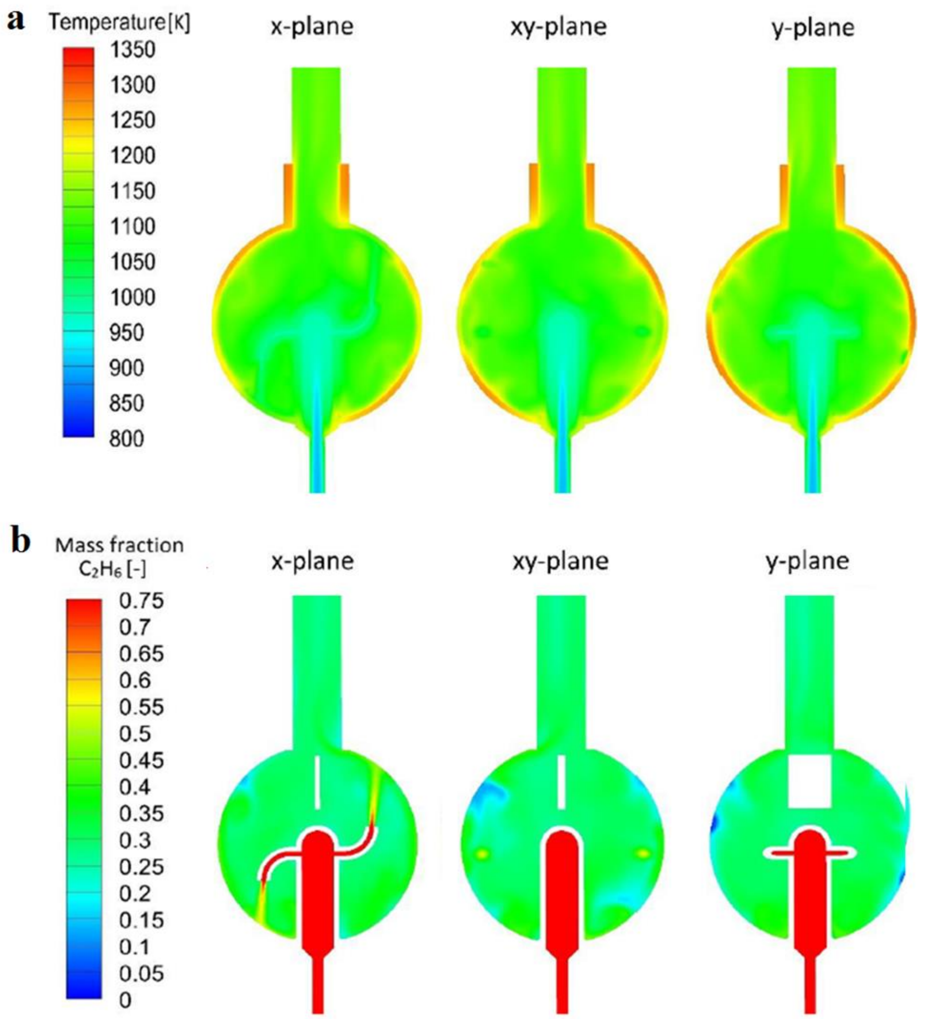

Figure S2. Cross section fields of temperature (**a**) and ethane mass fraction (**b**) for ethane cracking experiments in the JSR [1].

Internal Standard

In detail, from the peak areas of the TCD-channel, experimentally determined calibration factors and the known amount of nitrogen, the flows of hydrogen, methane, carbon oxides and C2 hydrocarbons are calculated. The calculated methane flow is subsequently used as internal standard for the flow determination of higher hydrocarbons in the FID-channels. In Figure S3, a schematic representation of the use of the internal standard is given in supporting information.

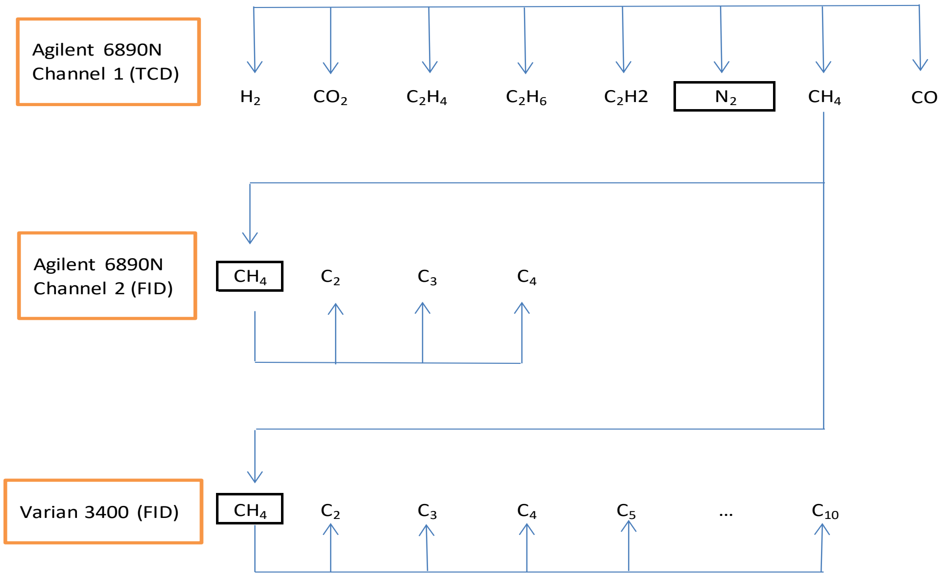

Figure S3. Schematic overview of the use of internal standards for quantitative composition analysis.

Coke Formation Mechanisms

The carbon deposition process during thermal cracking in the presence, or not, of steam, is a quite complex phenomenon. It has been extensively described in literature [2–5] that it mainly consists of three mechanisms; catalytic coke formation, coke growth from existing carbon layers onwards (pyrolytic) and gas phase coking, as illustrated in Figure S4.

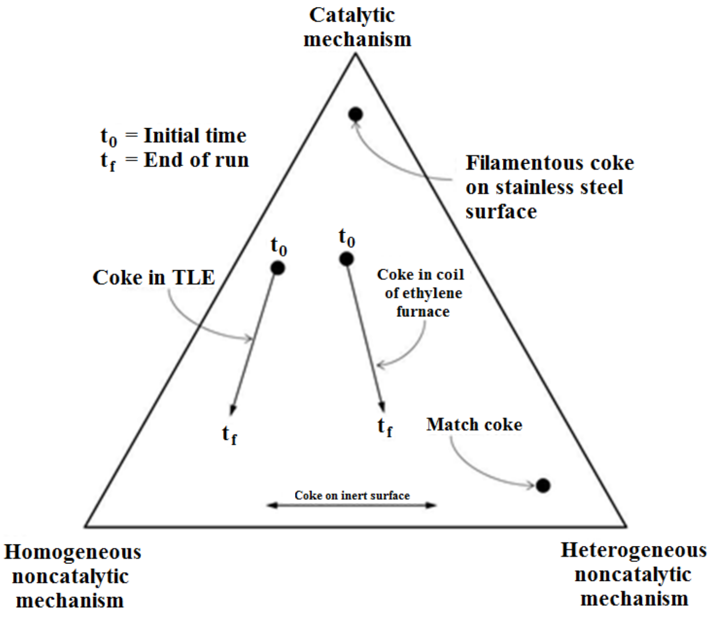

Figure S4. Comparison of the relative importance of the three main coking mechanisms [6].

Coupon Breakage

As mentioned in the manuscript one of the major problems with the industrial application of the Ti-base alloy is the breakages occurring on the coupons after cooling down. In Figure S5, an indication of the coupon thickness decrease after cracking and after application of the pretreatment A, but also of the slices thickness is given. The total fresh coupon thickness is around 1 mm. By addition of the

slice thickness and coupon thickness after cracking, it is clear that the thickness before breakage exceeded the 1.1 mm. This, due to the formation of thick oxides during cyclic coking and oxidation of Ti—almost 10% of the total coupon volume - making the coupon more brittle as material towards mechanical stresses. The latter leads to the formation of cracks on the alloy due to the mechanical stresses evolved especially during cooling down at temperatures lower than 1023 K.

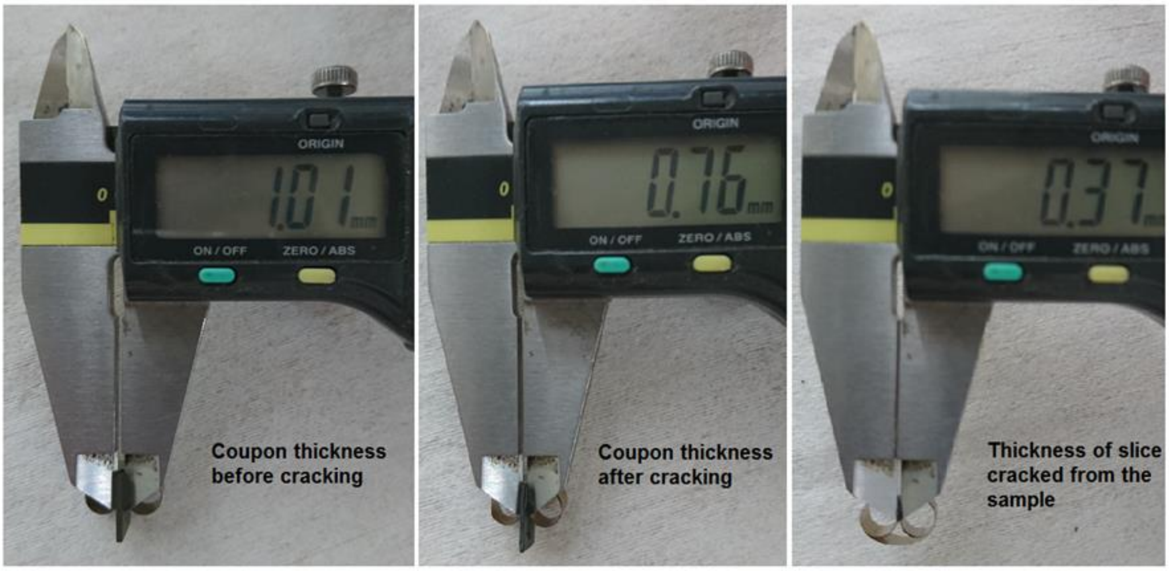

Figure S5. Thickness values of the Ti-base alloy before cracking (left), after cracking (middle) and of a broken slice (right), pretreated at 1173 K.

Another example of a cracked coupon after pretreatment B is illustrated in Figure S6. Here, the breakage is more pronounced occurring diagonally and leading to smaller coupon pieces. To illustrate more accurately the crack formed on the coupons and for a better understanding of the phenomenon, SEM and EDX analyses were performed.

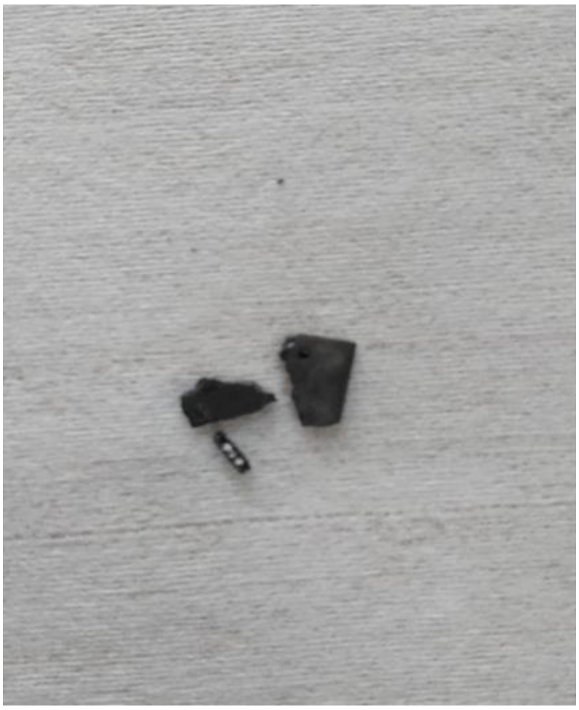

Figure S6. Broken Ti-base alloy coupon after cracking pretreated at 1273 K.

References

1. Reyniers, P.A.; Sarris, S.A.; Marin, G.B.; Van Geem, K.M. Computational Fluid Dynamic Design of Jet Stirred Reactors for Measuring Intrinsic Kinetics of Gas-Phase and Gas-Solid Reactions. *Int. J. Chem. Kinet.* **2016**, *48*, 556–569, doi:10.1002/kin.21016.
2. Kopinke, F.D.; Zimmermann, G.; Nowak, S. On the mechanism of coke formation in steam cracking—conclusions from results obtained by tracer experiments. *Carbon* **1988**, *26*, 117–124, doi:10.1016/0008-6223(88)90027-9.
3. Cai, H.; Krzywicki, A.; Oballa, M.C. Coke formation in steam crackers for ethylene production. *Chem. Eng. Process.: Process Intensific.* **2002**, *41*, 199–214, doi:10.1016/S0255-2701(01)00135-0.
4. Albright, L.F.; Marek, J.C. Mechanistic model for formation of coke in pyrolysis units producing ethylene. *Ind. & Eng. Chem. Res.* **1988**, *27*, 755–759, doi:10.1021/ie00077a006.
5. Lahaye, J.; Badie, P.; Ducret, J. Mechanism of carbon formation during steamcracking of hydrocarbons. *Carbon* **1977**, *15*, 87–93, doi:10.1016/0008-6223(77)90022-7.
6. Albright, L.F.; Marek, J.C. Mechanistic Model for Formation of Coke in Pyrolysis Units Producing Ethylene. *Ind. Eng. Chem. Res.* **1988**, *27*, 755.
